# Novel Therapeutics for Epstein–Barr Virus

**DOI:** 10.3390/molecules24050997

**Published:** 2019-03-12

**Authors:** Graciela Andrei, Erika Trompet, Robert Snoeck

**Affiliations:** Laboratory of Virology and Chemotherapy, Department of Microbiology and Immunology, Rega Institute for Medical Research, KU Leuven, 3000 Leuven, Belgium; erika.trompet@kuleuven.be (E.T.); robert.snoeck@kuleuven.be (R.S.)

**Keywords:** Epstein–Barr virus, antivirals, nucleoside analogues, nucleotide analogues, cellular targets

## Abstract

Epstein–Barr virus (EBV) is a human γ-herpesvirus that infects up to 95% of the adult population. Primary EBV infection usually occurs during childhood and is generally asymptomatic, though the virus can cause infectious mononucleosis in 35–50% of the cases when infection occurs later in life. EBV infects mainly B-cells and epithelial cells, establishing latency in resting memory B-cells and possibly also in epithelial cells. EBV is recognized as an oncogenic virus but in immunocompetent hosts, EBV reactivation is controlled by the immune response preventing transformation in vivo. Under immunosuppression, regardless of the cause, the immune system can lose control of EBV replication, which may result in the appearance of neoplasms. The primary malignancies related to EBV are B-cell lymphomas and nasopharyngeal carcinoma, which reflects the primary cell targets of viral infection in vivo. Although a number of antivirals were proven to inhibit EBV replication in vitro, they had limited success in the clinic and to date no antiviral drug has been approved for the treatment of EBV infections. We review here the antiviral drugs that have been evaluated in the clinic to treat EBV infections and discuss novel molecules with anti-EBV activity under investigation as well as new strategies to treat EBV-related diseases.

## 1. Introduction

The human γ-herpesviruses Epstein–Barr virus (EBV or human herpesvirus 4, HHV-4) is one of the most commonly contracted herpesvirus, infecting up to 95% of the adult human population. Primary EBV infection generally occurs during childhood and is usually asymptomatic. However, EBV infection in adolescence and early adulthood may lead to infectious mononucleosis in 35% to 50% of the cases. The symptoms of infectious mononucleosis (fatigue, fever, inflamed throat, swollen lymph nodes in the neck, enlarged spleen, swollen liver, rash) typically subside in 1 to 2 months, although the rates of chronic fatigue symptoms in adolescents following resolution of infectious mononucleosis are of 13%, 7%, and 4%, at 6, 12, and 24 months, respectively [1].

Similar to other herpesviruses, EBV establishes lifelong latency following primary infection. Most of the persons who carry EBV for a lifetime do not suffer from the viral infection because EBV infection is controlled by the immune system. However, EBV can also cause severe acute diseases and a range of life-threatening malignancies of lymphoid and epithelial cell origin under immunosuppressed conditions (congenital, in the context of HIV infections, or linked to the use of immunomodulatory drugs in transplantation and in autoimmune diseases). EBV is associated with the development of B-cell malignancies, such as Burkitt’s lymphoma and other non-Hodgkin’s lymphomas, Hodgkin’s lymphoma, central nervous system lymphomas, and post-transplant lymphoproliferative disorder (PTLD), AIDS-associated lymphoma as well as natural killer (NK), and T-cell lymphomas [2,3] (Table 1). It is also found in 100% of non-keratinizing nasopharyngeal carcinomas and has been linked sporadically to cancers of the gastrointestinal tract [4,5].

**Figure 1:**
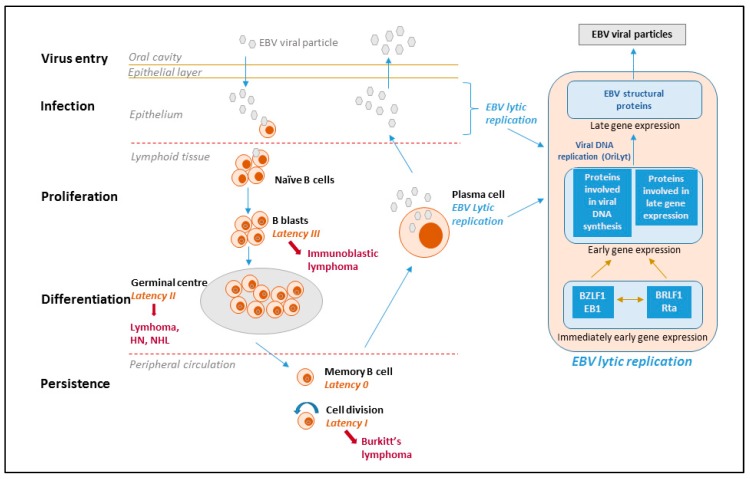
EBV life cycle, latency stages and derived lymphomas. The viral life cycle includes at least five different stages (virus entry, infection, proliferation, differentiation and persistence), and four of them are associated with EBV diseases. The virus is transmitted through the saliva and infects naïve B-cells in the oropharyngeal mucosa. During primary infection, EBV-infected naïve B-cells express the entire latency gene complex (10 proteins: EBV nuclear antigens (EBNAs), latent membrane protein (LMPs)) as well as EBV-encoded small RNAs (EBERs) and microRNAs. This is called type III latency and this form of latency activates the resting B-cells and drives them to proliferation and transformation. However, these cells are highly immunogenic and are rapidly eliminated by EBV-specific T cells. The virus is able to survive in B-cells because it downregulates its immunogenic proteins. EBV mimics antigen driven B-cell responses and similar to antigen-stimulated blasts, the EBV-infected B-cells enter the follicles, expand, and form germinal centers where they express only three viral proteins (type II latency). Finally, they exit the lymph node expressing only a single viral protein (EBNA1, which ensures that the viral genome divides with the cellular genome) (type I latency). The entry of EBV-infected cells into the peripheral blood results in the shutdown of all viral genes encoding for proteins; this is called latency 0 or latency program where no viral proteins are expressed. Resting memory cells, in which the virus is quiescent, are not attacked by the host immune system and are likely the sites of long-term persistence. Memory B cells occasionally divide to maintain stable number of cells and when a cell that is carrying the virus divides, the viral EBNA1 protein is expressed to allow the viral genome to replicate along with the cell. Memory B-cells may also undergo terminal differentiation into plasma cells and secrete antibodies. If such a cell contains the virus, the EBV lytic program is activated and the infectious virus released from the plasma cells can infect epithelial cells, where the virus can replicate and be shed at high amounts and then be transmitted to other hosts. With the exception of latency type 0, each latency state is found in specific types of EBV-associated malignancies.

The EBV life cycle displays two distinct phases, i.e., lytic and latent (Figure 1). EBV preferentially infects B-cells via the CD21 receptor but also infects epithelial cells as well as T- or NK-lineage cells at a lower frequency. The virus undergoes lytic replication in epithelial cells and establishes lifelong latency in circulating memory B-lymphocytes, reactivating periodically from latency [4,6]. During lytic infection, the full repertoire of viral gene expression takes place and progeny virus is produced. The two key EBV immediate-early lytic genes (i.e., *BZLF1* and *BRLF1*) encode transactivators that activate viral and certain cellular promoters, leading to an ordered cascade of viral gene expression: early gene expression and genome replication followed by late gene expression. Virions produced during lytic replication in permissive epithelial cells allow the dissemination of viral particles within and among hosts.

**Figure 2:**
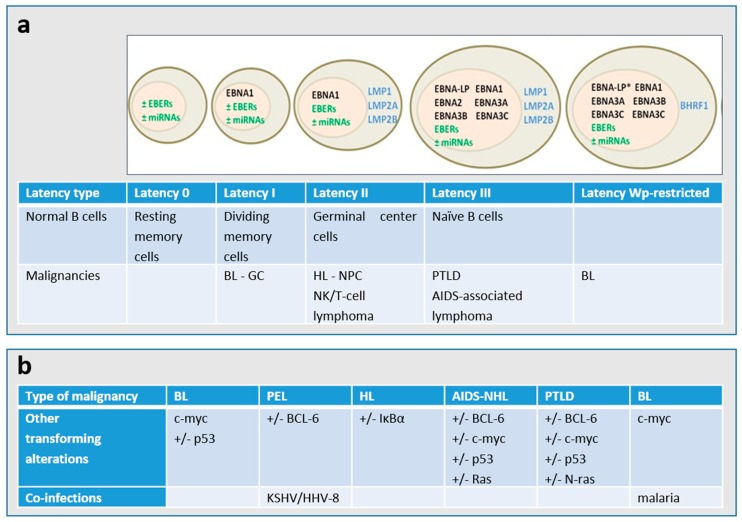
(**a**) Patterns of gene expression during EBV latency. The majority of the endemic BL presents a latency I type and carry a wild-type transformation-competent EBV genome and express only the Epstein–Barr nuclear antigen 1 (EBNA1) from the *EBNA1*-specific latent promoter Qp, non-coding EBERs (Epstein–Barr virus-encoded small RNAs) and several microRNAs (miRNAs). Around 15% of BL endemic tumors, the so called Wp-restricted BLs, carry an *EBNA2* gene-deleted genome and express EBNA1, -3A, -3B, and-3C and the viral Bcl2 homologue BHRF1 from the Wp latent promoter [2,6]. * The EBNA-LP gene is partially deleted in the Wp-restricted latency. A major type of latency in EBV-associated malignancies is latency II, in which the latent membrane proteins LMP1, LMP2A, and LMP2B are expressed in addition to the Latency I genes. The entire EBV latency gene complex, which consists of several EBNA proteins, LMP1, LMP2A, LMP2B, EBERs, and miRNAs are expressed in the type III latency. (**b**) The cellular genetic alterations and/or co-infections are known to occur in the different types of EBV-associated malignancies. PEL: primary effusion lymphoma; HL: Hodgkin lymphoma; BL: Burkitt lymphoma; NHL: non-Hodgkin lymphoma; PTLD: post-transplant lymphoproliferative disorder; NPC: nasopharyngeal carcinoma; GC: gastric carcinoma.

In the course of latency, the virus expresses only a limited number of genes required for the maintenance of the viral genome (as an episome in the nucleus) and evasion of the host immune system. Based on the expression of latent genes, EBV latency is classified in different types (Figure 2A) [7]. It is worth noting that the Latency 0, I, II, III nomenclature represents a snapshot of gene expression. EBV latency ranges from latency 0 (without EBV antigen expression, as observed in circulating memory B-cells in healthy persons) to latency III (in which all nuclear proteins (EBNAs -1,-2, -3A, -3B, -3C, and -LP) and 2 membrane proteins (LMP1 and LMP2) are expressed together with 2 small RNAs (EBERs), as found in PTLD. Some tumors may not fall into one of these patterns of latency and furthermore, immunohistochemistry may indicate heterogeneity of expression within a single biopsy. In addition, cellular genetic alterations and/or co-infections occur in EBV-associated malignancies (Figure 2B). Although latency programs predominate in EBV-driven tumors, lytic viral replication is also of pathogenic importance [8,9].

## 2. Why Is There No Antiviral Drug Approved for the Treatment of EBV Infections?

Nucleoside (i.e., acyclovir (ACV), penciclovir (PCV), ganciclovir (GCV), and its oral prodrugs; valacyclovir (VACV), famciclovir (FAM), and valganciclovir (VGCV), respectively), nucleotide (i.e., cidofovir (CDV)), and pyrophosphate (i.e., foscavir (foscarnet sodium, PFA)) analogues are approved for the treatment of herpes simplex virus 1 (HSV-1) and 2 (HSV-2), varicella-zoster virus (VZV), and/or human cytomegalovirus (HCMV) [10,11]. In some European countries, brivudin (BVDU) is approved for the therapy of HSV-1 and VZV associated diseases. Although some of these antiviral agents proved to be effective inhibitors of EBV replication in vitro and were used experimentally [11,12,13], none of them received approval by the FDA (Food and Drug Administration) or EMA (European Medicines Agency) for treatment of EBV infections.

In 2005, Gershburg and Pagano proposed three main explanations for the lack of an anti-EBV drug [14]. First, the difficulty in diagnosing infectious mononucleosis may be, at least in part, responsible for the lack of success in the development of a drug to treat EBV-associated infections. While EBV infects most persons at the age of 30, only a few of them suffer from infectious mononucleosis (usually those who acquired the infection in the twenties). The infectious mononucleosis symptoms are subtle in onset and the disease has a long incubation time (4–6 weeks), resulting in a late diagnosis, in contrast to infections caused by the α-herpesviruses HSV (i.e., herpes labialis) or VZV (i.e., chickenpox).

Second, antivirals should be achieving high concentrations in the oropharynx where EBV is released at high titers. Although acyclovir was shown to significantly reduced EBV shedding in the oropharynx when administered intravenously and orally, virus release resumed at the initial level within 3 weeks of cessation of the treatment [15,16]. Maybe the most important reason for the failure of antivirals for infectious mononucleosis therapy can be ascribed to the fact that the symptoms and signs of the disease are not the consequences of viral replication but the immunological response to EBV-infected B-cells that circulate in the blood and infiltrate the tissues of different organs. Infectious mononucleosis is characterized by atypical lymphocytosis due to the massive cell-mediated immune response against viral-infected B-lymphocytes. Thus, antivirals in combination with immunomodulatory drugs (such as corticosteroids, used empirically by physicians to treat infectious mononucleosis) might be effective. However in a multicenter, double-blind, placebo controlled study, prednisolone administered with acyclovir for treatment of infectious mononucleosis inhibited oropharyngeal EBV replication without affecting duration of clinical symptoms or development of EBV-specific cellular immunity [16].

## 3. Medical Need for Anti-EBV Therapeutics Targeting Lytic Replication

Primary EBV infection is usually asymptomatic but some patients develop infectious mononucleosis, which can have mild symptoms (i.e., fever, sore throat and lymphoadenopathy) or be fatal in the immunocompromised hosts. Furthermore, primary EBV infection with or without infectious mononucleosis may lead to complications (such as autoimmune hemolysis, airway obstruction from enlarged tonsils, splenic rupture, encephalitis, severe hepatitis and myocarditis), which are primarily a consequence of the immunopathological responses to the virus. Other rare but serious complications (such as agranulocytis and aplastic anemia) may occasionally arise in healthy patients. The mainstay for treatment of complications related to infectious mononucleosis are corticosteroids. Although the role of antivirals in the management of severe infectious mononucleosis complications is debatable based on case series, physicians may consider the use of antiviral agents in severe manifestations of EBV infections. Even though the pathogenesis of infectious mononucleosis is primarily immune mediated, the severity of EBV-associated hepatitis was shown to be related to a high viral burden [17,18]. Therefore, the use of specific antivirals is expected to alleviate the symptoms of EBV-related complications found in infectious mononucleosis.

EBV lytic replication is not only directly associated with infectious mononucleosis but also with chronic active EBV infection (CAEBV) and oral hairy leukoplakia. CAEBV is rare in USA and Europe but occurs more frequently in Asia and South America. CAEBV is a lymphoproliferative disorder with markedly elevated levels of EBV-specific antibodies and high viral loads. It is often a fatal disorder characterized by chronic or recurrent infectious mononucleosis-like symptoms persisting for a long time and by an unusual pattern of anti-EBV antibodies. Life-threatening CAEBV complications include virus-associated hemophagocytic syndrome, leukemia and lymphoma of T/NK-cell lineages [19]. Intragenic EBV deletions that reactivate the lytic cycle by upregulating the expression of immediately early genes were linked to avert viral production and promotion of lymphomagenesis [20]. Immunomodulatory agents (such as interferon-α and interleukin-2), antivirals (including acyclovir, ganciclovir and vidarabine), chemotherapeutic agents, cell therapy using EBV-specific cytotoxic T lymphocytes, and hematopoietic stem cell transplantation have been used for treatment of CAEBV but with limited success [21].

Post-transplant lymphoproliferative disorder (PTLD) is a serious and often fatal complication following solid organ transplantation, being EBV a major risk factor for PTLD in this group of patients as 30% to 50% of EBV-naïve patients that seroconvert are diagnosed with PTLD [22,23]. PTLD is also a life threatening disorder following allogeneic hematopoietic stem cell transplant (HSCT), caused by drug-induced reduced immune surveillance leading to an uncontrolled proliferation of lymphocytes. PTLD incidence has increased significantly during the last two decades due to several reasons, including a growing number of transplant activities, increasing age of donors and recipients, use of new and potent immunosuppressive agents, introduction of haplo-identical HSCT, increased awareness of the disorder and improved diagnostic tools [24]. A sustained persistence of high viral load and an elevated viral load have been associated with increased risk of PTLD [25,26]. Antiviral therapy has not been regarded as useful in PTLD because the virus is latent. However, prophylactic administration of antiviral drugs resulted in reduce incidence of PTLD [27,28,29]. Antiviral agents may have a role in preventing PTLD as high level of EBV DNA in the blood of solid organ transplant recipients has been shown to predict PTLD. Most cases of early onset PTLD (occurring during the first year following transplantation) are associated with recent EBV infection. Late-onset lymphomas occurring after the first year of transplantation are less likely to be associated with EBV.

Treatment strategies aimed at suppressing the expression of lytic proteins should be helpful for controlling early stages of EBV-associated malignancies as EBV lytic infection was shown to contribute to lymphoproliferative disease [8,30]. The EBV IE proteins BZLF1 and BRLF1 contribute to IL-6 secretion in lytically infected cells promoting early lymphoproliferative disease [31]. IL-6 is a cytokine known to play a crucial role in the maintenance of immune functions, stimulation of differentiation of hematopoietic cells and perpetuation of inflammation but it is an important factor in a variety of hematological and epithelial malignancies. IL-6 acts through paracrine and autocrine mechanisms to promote cell survival and induces the signal transducer and activator of transcription 3 (STAT3). Therefore, it is not surprising that viruses (such as EBV) capable of infecting both epithelial and lymphoid cells, have mechanisms to induce IL-6 expression. Also, lytically infected cells induce the expression of cellular and viral IL-10 [32], allowing B-cells to grow more efficiently, and of VEGF contributing to angiogenesis in both B-cell and epithelial malignancies [33].

## 4. Antivirals Against EBV Evaluated in The Clinic

### 4.1. Nucleoside Analogues (Acyclovir, Valacyclovir, Ganciclovir, and Valganciclovir)

As acyclovir and ganciclovir inhibit EBV in vitro [11,34,35], these drugs and their oral prodrugs were evaluated for suppression of EBV reactivation during immunosuppression. PTLD incidence in lung and heart-lung transplant EBV-seronegative recipients was reduced by antiviral prophylaxis with acyclovir, valacyclovir or ganciclovir [28]. The incidence of this lymphoproliferative disorder was analyzed before 1996 (historic group) and between 1996 and 2000 (group receiving antiviral prophylaxis) to compare the impact of long-term antiviral prophylaxis on PTLD development in EBV-seronegative recipients. None of the EBV-seronegative recipients receiving continuous antiviral prophylaxis developed PTLD while in the historic group, PTLD developed in 4.2% of the patients.

The effects of ganciclovir and valganciclovir prophylaxis on EBV viral load were evaluated in a group of EBV-naïve pediatric renal transplant recipients (R-) who had received a graft from an EBV-positive donor (D+) and therefore at risk to develop EBV primary infection [27]. Over the first year post-transplantation, antiviral prophylaxis with ganciclovir or valganciclovir resulted in a significant decreased incidence of EBV primary infection: 9/20 (45%) in the prophylaxis group had a primary EBV infection versus 8/8 (100%) in the non-prophylaxis group. Antiviral prophylaxis afforded a significantly lower EBV viral load while the type or intensity of immunosuppressive therapy did not affect the incidence of EBV primary infection or the level/persistence of viral load.

The impact of antiviral drugs used to prevent HCMV disease was investigated in a monocentric retrospective cohort of 73 adult kidney or kidney-pancreas EBV-seronegative recipients, transplanted between January 2000 and January 2016 [36]. Thirty-seven (50.7%, prophylaxis group) received valacyclovir or valganciclovir for 3–6 months and 36 (49.3%, no-prophylaxis group) received no antivirals with mean follow-up times of 69 months (prophylaxis group) and 91 months (no-prophylaxis group). Prophylaxis delayed primary infection at 100 days (43% versus 84%) as determined by monitoring EBV viral load. Early PTLD incidence did not differ between groups but EBV-related neoplasia incidence was significantly lower in treated patients (no cases observed) than in the no-prophylaxis group (six neoplasia cases reported). Despite a weak level of evidence, antiviral prophylaxis could prevent late onset PTLD.

In a cohort of pediatric liver transplant recipients, treatment with intravenous ganciclovir did not change the proportion of patients with reduction in EBV load at 8 weeks and 1 year after detection of EBV viremia [37]. This retrospective study performed in Norway from 2002 until 2015 included 38 patients with EBV viremia and 32 of them were treated with intravenous ganciclovir for a median of 22 (21–38) days. Short time from transplantation to viremia, younger age at transplantation and lack of EBV seroconversion prior to transplantation were significant predictors of chronic EBV viremia.

Valganciclovir suppressed EBV reactivation in a group of 29 patients under immunosuppression with alemtuzumab (a humanized monoclonal antibody against CD52 expressed on all B- and T-lymphocytes), which predisposes to HCMV and EBV reactivation [38]. Plasma EBV DNA load was quantified in 29 patients (258 samples with a median of seven specimens per patient). In 24 of the patients, no quantifiable EBV DNA was detected while five patients (17%) had EBV reactivation that dropped spontaneously in four cases. One patient, who had also received previously another potent T-cell suppressing drug (fludarabine), developed EBV-positive Hodgkin lymphoma.

The efficacy and safety of valganciclovir without immunosuppression decrease was assessed in a group of children undergoing liver transplantation who showed sustained EBV DNA in their blood [29]. Undetectable viral load was observed in 20/42 (47.6%) of patients under prolonged antiviral therapy (median 8 months), 60% of whom maintained response to therapy. However, the results of this study should be interpreted with cautious because of the lack of a control group.

The effects of valganciclovir on oral EBV shedding were evaluated in a randomized, double blind, placebo-controlled study [39]. Twenty-six men were included and all participants self-identified as men who have sex with men, and 16 participants (62%) were HIV-1 infected. They received oral valganciclovir or daily placebo for 8 weeks, followed by a 2-week “washout period” and then 8 weeks of the alternative treatment. Valganciclovir significantly reduced the proportion of days with EBV detected from 61.3% to 17.8% and the quantity of virus detected by 0.77 logs, which warrants further investigations into the impact of valganciclovir on EBV-associated diseases.

### 4.2. Nucleotide Analogues

The nucleotide analogue cidofovir (CDV) is a broad spectrum anti-DNA virus agent. The compound is approved for the treatment of retinitis in AIDS patients but is used off-labeled to treat several infections caused by DNA viruses [40]. Besides its recognized antiviral properties, the drug is also known for its antiproliferative effects [41].

Successful treatment of locally recurrent EBV-associated nasopharyngeal carcinoma using the antiviral agent cidofovir was reported in two patients [42]. Further, injection of cidofovir into the tumor tissue of EBV-positive nasopharyngeal carcinoma xenografts in nude mice suppressed tumor growth [43]. In combination with the ribonucleotide reductase inhibitors hydroxyurea and didox (3,4-dihydroxybenzohydroxamic acid), cidofovir-induced apoptosis in EBV-transformed epithelial cells and in EBV-positive nasopharyngeal carcinoma xenografts was augmented [44]. Cidofovir decreased EBV oncoproteins and enhanced the radiosensitivity in EBV-related malignancies (Burkitt’s lymphoma and nasopharyngeal carcinoma) [45].

### 4.3. Pyrophosphate Analogues

Foscarnet, an inorganic pyrophosphate analogue, is a direct inhibitor of herpesvirus DNA polymerases. It blocks the pyrophosphate-binding site and prevents cleavage of pyrophosphate from deoxynucleoside triphosphates. Foscarnet, a non-competitive inhibitor of viral DNA polymerases, does not incorporate into the growing viral DNA and is ~100-fold more active against viral than cellular enzymes. Although the drug has activity against all human herpesviruses, including EBV, foscarnet is approved for treatment of HCMV retinitis in AIDS patients and for the therapy of acyclovir-resistant HSV infections in immunocompromised patients. It has also been used for therapy of ganciclovir-resistant HCMV infections due to mutations in the UL97 protein kinase (PK). Its safety and efficacy for the treatment of other herpesvirus infections has not yet been established [10]. The successful use of foscarnet to manage a persistent EBV infection was occasionally reported. Foscarnet in combination with immunoglobulins was successful to control the persistent EBV infection in a lung transplant patient that showed clinical improvement of PTLD following reduction in immunosuppression intensity [46]. This patient required treatment with an antiviral drug and immunoglobulins since restoration of the cellular immunity improved PTLD but was ineffective against controlling the EBV infection. Regression of EBV-associated lymphoproliferative disorders in two AIDS patients during therapy with foscarnet has also been described [47].

## 5. Anti-EBV Compounds Under Investigation

### 5.1. Inhibitors of EBV Protein Kinase BGLF4

Maribavir (MBV) is an investigational oral benzimidazole L-riboside with significant activity against both HCMV and EBV but no other human herpesviruses [48,49]. Maribavir has fewer adverse side effects and is more specific compared to anti-HCMV drugs that target the viral DNA polymerase. Unlike nucleoside and nucleotide analogues, maribavir’s inhibitory effects are mainly produced through inhibition of the HCMV and EBV PKs [50]. This compound selectively inhibits the HCMV UL97 PK (as determined by direct inhibition of kinase activity) and single point mutations in the UL97 gene confer maribavir resistance in HCMV [51]. UL97 is a serine/threonine-specific PK playing an important role in HCMV egress. UL97 is necessary for the phosphorylation of several viral and cellular proteins in HCMV infected cells [50,52]. In vitro and in vivo UL97 mutations selected under ganciclovir and maribavir were found to be distinct and to confer no cross-resistance [53,54], while partial cross-resistance between ganciclovir and cyclopropavir, a methylenecyclopropane nucleoside analog active against HCMV was observed [51,55]. However, Chou and colleagues [56] reported UL97 kinase mutations either found in ganciclovir-treated subjects or after propagation under cyclopropavir in vitro with moderate- to high-level resistance to all three drugs. Low levels of resistance (two- to three-fold) to maribavir can also arise due to mutations in the HCMV *UL27* gene. Although the function of UL27 is unknown, it does not appear to be a direct target for maribavir.

The efficacy of maribavir prophylaxis for prevention of HCMV disease in recipients of allogeneic stem-cell transplants was evaluated in phase three: double blind, placebo-controlled, randomized trials [57,58]. The clinical development of maribavir for the management of HCMV infections is currently on hold because the drug failed to meet the primary endpoint (prevention of HCMV disease) in recipients of allogeneic stem-cell transplants although several critics on the study design were raised [59].

Maribavir exhibits also marked activity against EBV, having a unique dual effect against EBV: inhibition of viral DNA replication and of virus transcription [14,60]. In contrast to HCMV, the activity of maribavir against EBV could not be ascribed to direct inhibition of the EBV PK BGLF4. In fact, maribavir treatment was shown to inhibit the phosphorylation of the EBV DNA polymerase processivity factor *BMRF1* [49]. Unlike acyclovir that has little effect on EBV RNAs, maribavir inhibits the expression of multiple RNAs. Furthermore, the inhibitory profile of maribavir transcripts appeared to be similar to that produced by an EBV mutant in which PK expression and activity were knocked out [61], suggesting that maribavir largely affects EBV transcript levels through inhibition of BGLF4 although the drug does not directly affects the EBV PK [62]. Considering that EBV BGLF4 has at least 20 viral targets, maribavir may also affect downstream targets indirectly.

### 5.2. Inhibitors of EBV DNA Polymerase

The novel l-dioxolane thymidine analog, 1-[(2S,4S-2-(hydroxymethyl)-1,3-dioxolan-4-yl]5-vinylpyrimidine-2,4(1H,3H)-dione, or HDVD, proved active against HSV-1, KSHV, EBV, as well as murine herpesvirus 68 (MHV-68) [35]. The compound weakly inhibited replication of HSV-2, VZV and herpesvirus saimiri (HVS), and no antiviral activity was found against HCMV and rhesus rhadinovirus (RRV). Thus, its antiviral activity spectrum differed from that of the related compound brivudin (which is known for its potent activity against HSV-1 and VZV). However, similar to brivudin, characterization of HDVD-resistant viruses indicated that the viral thymidine kinases (TKs) of HSV-1, MHV-68, and HVS were required for activation of the compound. Oral treatment with HDVD and brivudin was assessed in an intranasal model of MHV-68 infection in BALB/c mice. HDVD treatment, in contrast to brivudin treatment, resulted in a reduction in viral DNA load and diminished viral gene expression during acute viral replication in the lungs compared to untreated controls. The valyl ester prodrug of HDVD (USS-02-71-44) was more effective in preventing the latent infection in the spleen than HDVD [35]. Studies on mechanisms of resistance of various nucleoside derivatives indicated that pyrimidine nucleoside derivatives are phosphorylated by the γ-herpesvirus TK and purine nucleosides are preferentially activated by the γ-herpesvirus PK [13], consistent with previous findings showing that the EBV-encoded PK, but not the TK, is required for ganciclovir and acyclovir inhibition of lytic viral production [63].

Two thionucleoside derivatives, KAY-2-41 and KAH-39-149, displayed effective in vivo (MHV-68 mouse model) antiviral efficacy and potent and selective in vitro anti-EBV activity [34]. The compounds also proved active against HSV and VZV. KAY-2-41- and KAH-39-149-resistant HSV and MHV-68 harbored mutations in the viral TK though these mutations conferred only low levels of resistance to KAY-2-41 and KAH-39-149 compared to other TK-dependent drugs. Antiviral assays in HeLa TK-deficient cells showed a lack of KAY-2-41 and KAH-39-149 activities against HSV TK-deficient mutants. Furthermore, enzymatic assays showed the ability of HSV-1 and VZV TK, and cellular TK1 and TK2 to recognize and phosphorylate KAY-2-41 and KAH-39-149, demonstrating that the compounds depend on both viral and host TKs to exert antiviral activity. KAH-39-149 proved superior to KAY-2-41 in a mouse model of γ-herpesvirus infection, highlighting their potential as antiviral therapeutics against EBV.

Various methylenecyclopropane nucleoside (MCPN) analogues proved active against several herpesviruses in cell culture and animal models [64,65,66,67,68]. The first series of MCPN analogues had a single hydroxymethyl group on the cyclopropane ring mimicking the 5′ hydroxyl moiety in deoxyribonulceosides. Compounds from this series, similar to acyclovir, block further strand elongation once incorporated into DNA because of the lack of a counterpart to the 3′ hydroxy group. The second generation of MCPN analogues are dihydroxymethyl derivatives, and similar to ganciclovir, have a second hydroxyl group that can be recognized by the DNA polymerase allowing further strand elongation. Cyclopropavir, the most active compound of this class, displays good antiviral activity against HCMV, murine CMV, EBV, HHV-6 and HHV-8 and its prodrug 6-deoxycyclopropavir has shown good activity when administered orally [69]. The mechanism of action of cyclopropavir against CMV is complex involving both the inhibition of DNA synthesis and the UL97 PK [70]. Analogues of the first series with a single hydroxymethyl group, such as (S)-synguanol exhibit a broader spectrum of antiviral activity encompassing hepatitis B and HIV. More recently, monohydroxymethyl and dihydroxymethyl analogues with 6-ether and –thioether moieties have also been reported to be active against several herpesviruses, including EBV. Some of these analogues exhibit a broader spectrum of activity than cyclopropavir inhibiting also HSV-1, HSV-2 and VZV [66]. The activity of these compounds was dependent on the HCMV UL97 PK but was relatively independent from HSV TK activity. These data point to a different mechanism of action of these analogues from that of cyclopropavir and suggest that they can eventually be used as broad-spectrum anti-herpesvirus agents.

*N*-methanocarbathymidine, a conformational locked nucleoside analogue, proved active against α-herpesviruses, γ-herpesviruses and orthopoxviruses [71]. The antiviral activity of this compound is dependent on the activation by viral TKs. The drug inhibits viral DNA synthesis once it is activated by the viral TKs. The compound proved also effective in animal models of orthopoxvirus and HSV infection [72,73] and there is an ongoing Phase I trial in healthy volunteers [10] to evaluate its safety.

Several acyclic nucleoside phosphonates (ANPs), including cidofovir derivatives, inhibited with high potency and selectivity the replication of EBV and other γ-herpesviruses [12] Notable, cyclic prodrugs of ANPs exhibited reduced activities against the EBV strain P3HR-1, but not against the EBV strain Akata. Metabolism studies with cidofovir and its cyclic form (cyclic-cidofovir) revealed that these differences were attributable to an altered drug metabolism in P3HR-1 cells after EBV reactivation, i.e., to a reduced hydrolysis of cyclic-cidofovir by cyclic CMP phosphodiesterase [12]. Altered cyclic AMP levels in P3HR-1 cells implied a competitive inhibition of the phosphodiesterase by this cyclic nucleotide. Cidofovir and its 5-aza derivative (HPMP-5azaC) emerged as highly effective inhibitors of murine γ-herpesvirus replication and dissemination in a mouse model [12].

Brincidofovir (CMX-001) is the orally bioavailable form of cidofovir. This alkoxyalkyl ester prodrug of cidofovir has the same in vitro broad-spectrum antiviral activity as cidofovir but with an activity up to 1000-fold higher compared with cidofovir because of higher intracellular levels of cidofovir-diphosphate [74]. Besides its enhanced antiviral activity, brincidofovir is not nephrotoxic, because, in contrast to cidofovir, brincidofovir is not a substrate of the human organic anion transporter 1 enzyme located in the proximal renal tubule. Despite the promising preclinical data reported for brincidofovir, a Phase III trial evaluating its use for the prevention of HCMV disease in seropositive allogeneic hematopoietic stem cell transplant patients delivered disappointing results slowing down its progress to the market. In this trial, named SUPPRESS, increased HCMV disease was reported in the brincidofovir group compared to the placebo arm possibly related to the misdiagnosis of brincidofovir gastrointestinal disease as graft-versus host disease, which was treated with corticosteroids [75]. Following these findings, HCMV trials were suspended but adenovirus trials are ongoing and an IV formulation is in development. No trials are foreseen for evaluating this drug for EBV-associated diseases [10].

### 5.3. Inhibitors of EBV Nuclear Antigen 1 (EBNA1)

The EBV-encoded nuclear antigen 1 (EBNA1) is a versatile protein with functions in the maintenance, replication, and segregation of the EBV genome and represents an attractive therapeutic target to treat EBV-associated malignancies. This protein is express in all EBV latency types except for latency 0. The replication and persistence of the EBV episomal genome in latently infected cells primarily depend on the binding of EBV-encoded nuclear antigen 1 (EBNA1) to the cognate EBV oriP element.

Considerable efforts have been done the last years in the design or identification of inhibitors of EBNA-1 to decrease its expression or interfere with its functions. The salient features of EBNA-1, its functional domains and advances in the development of EBNA-1 inhibitors have been recently reviewed in detail [76]. For example, Lee and colleagues [77] characterized EBNA1 small molecule inhibitors (H20, H31) and their underlying inhibitory mechanisms. H20 fits into a pocket in the EBNA1 DNA binding domain (DBD) as predicted by in silico docking analyses but H20 did not significantly affect EBNA1 binding to its cognate sequence. A limited structure-relationship study of H20 allowed the identification of H31, a hydrophobic compound, as an EBNA1 inhibitor. H31 inhibited EBNA1-dependent oriP sequence-specific DNA binding activity, but did not affect sequence-nonspecific chromosomal association. H31 repressed the EBNA1-dependent transcription, replication, and persistence of an EBV oriP plasmid, consistent with the inhibition of EBNA1 binding activities. Importantly, H31 produced gradual loss of EBV episome and selectively delayed the growth of EBV-infected lymphoblastoid cell lines or Burkitt’s lymphoma cells. Thus, inhibition of EBNA1-dependent DNA binding by H31 decreased EBNA1-dependent transcription and persistence of EBV episome in EBV-infected cells. Screening approaches also identified molecules that could block EBNA1-DNA binding, EBNA1-oriP transactivation, EBNA1 linking regions. Also, inhibitors based on truncated peptides from EBNA1 dimeric interface were described confirming the “druggability” of EBNA1 for the treatment of EBV-related cancers [78].

Computational identification and structural characterization of EBNA1 binding pockets, likely to accommodate ligand molecules (i.e., “druggable” binding sites) were validated by docking against a set of compounds previously tested in vitro for EBNA1 inhibition (PubChem AID-2381) [79]. Assessments of pocket druggability were performed by induced fit docking and molecular dynamics simulations paired with binding affinity predictions by Molecular Mechanics Generalized Born Surface Area calculations for a number of hits belonging to druggable binding sites. These investigations established EBNA1 as a target for drug discovery, and provided the computational evidence that active AID-2381 hits disrupt EBNA1:DNA binding upon interacting at individual sites. Cullinan Oncology is developing a novel EBNA1 inhibitor, VK-2019 (that binds to EBNA1 and inhibits EBNA1 DNA binding activity), discovered by the Wistar Institute. There is currently a Phase 1–2a clinical trial (https://clinicaltrials.gov/ct2/show/NCT03682055), open-label, dose escalation and expansion, first-in-human clinical study to evaluate the safety and tolerability, pharmacokinetics, pharmacodynamics and preliminary efficacy of VK-2019.

## 6. Cellular Targets

An alternative strategy to direct acting antivirals designed to target a step of the viral replicative cycle, cellular proteins that are indispensable for viral replication may serve as novel targets to specifically hamper virus replication. Classical antiviral agents are active against a small number of viruses and resistance development is considered a hallmark of their specificity. In contrast, antivirals targeting cellular proteins essential for viral replication are expected to be active against a broader spectrum of viruses because replication of various unrelated viruses may involve the same cellular proteins. Further, antivirals targeting cellular events are expected to select less rapidly drug-resistant viral mutants than antivirals acting on viral proteins. Besides, they should remain active against viral mutants resistant to conventional antiviral agents. Yet, one of major drawbacks of targeting cellular proteins might be increased cytotoxicity and side effects.

As cellular topoisomerases I and II (Topo I and II) are essential for γ-herpesvirus lytic DNA replication [80,81], certain Topo I and II inhibitors may be considered as potential antivirals against EBV infection [82]. Topo II inhibitors are classified in two categories: Topo II poisons that target the topoisomerase-DNA intermediate and Topo II catalytic inhibitors that disrupt the turnover of the enzyme [83]. Topoisomerase II poisons include etoposide and doxorubicin, which are used as antitumor drugs and although they were shown to inhibit KSHV replication and virion production, as expected, they exhibited considerable toxicities [83]. In contrast, Topo II catalytic inhibitors, encompassing novobiocin, merbarone and rutamarin, exhibited antiviral activities against human γ-herpesviruses with minimal toxicities [83]. In particular, (+)rutamarin showed the highest selectivity (SI > 63) among the Topo II inhibitors tested and was able of inhibiting EBV DNA replication and virus production with little adverse effects on cell proliferation [82]. Therefore, rutamarin may be considered as a safe drug with potential for the treatment of human diseases associated with EBV infection.

Verdinexor belongs to a new class of novel small molecules known as SINE (Selective Inhibitors of Nuclear Export) compounds. These compounds covalently bind and block the nuclear export protein XPO1, leading to sequestration of XPO1-dependent proteins in the cell nucleus. Verdinexor showed various levels of efficacy against opportunistic viruses affecting immunocompromised individuals [84]. It was effective in inhibiting EBV replication in Akata cells, with 50% effective concentrations (EC_50_) of 50 nM and a selectivity index of 7. The efficacy of verdinexor could be explained by the dependence of the viral protein SM (adaptor protein involved in the nuclear-cytoplasmatic export of EBV mRNAs during lytic replication) on XPO1-mediated nuclear export. By blocking nuclear export, verdinexor prevents shuttling of EBV mRNAs to the cytoplasm for translation.

Several cellular protein kinase inhibitors have been tested for anti-herpesvirus efficacy as there is abundant evidence that host cellular protein kinases, and the downstream pathways that they control, play a crucial role in herpesvirus infections [85]. The success of mammalian target of rapamycin (mTOR) inhibitors in reducing HCMV disease in transplant patients may encourage further studies on the potential of cellular protein kinase inhibitors for therapy of herpesvirus-associated diseases [86,87,88]. Recently, everolimus was shown to delayed and suppress DNA synthesis, spread of the infection, and alleviated cytomegalovirus infection [89].

## 7. Medicinal Plants

A number of compounds isolated from medicinal plants are known to inhibit EBV lytic replication. Among them, *Andrographis paniculata*, commonly used to treat a range of illnesses, including bacterial infections, inflammations and high blood pressure, was shown to inhibit transcription of EBV IE genes and the production of EBV virions [90]. The diterpenoid andrographolide present in *A. paniculata* is important because of its anti-inflammatory, antithrombotic, anticancer and anti-immunostimulatory activities. Furthermore, andrographolide showed antiviral activity not only against EBV but also against other viruses including HIV [91,92], influenza virus, SARS [93] and HSV-1 [92,94]. It is currently unknown how andrographolide inhibits the transcription of EBV *BRLF1* and *BZLF1* genes but it is plausible that andrographolide inhibits signaling pathways that activate the transcription of EBV IE genes [90].

The ethyl acetate subfraction F3 obtained from *Polygonum cuspidatum* roots and its major component (i.e., emodin) were shown to inhibit EBV lytic cycle [95]. *P. cuspidatum* is a Chinese herbal medicine commonly used for the treatment of atherosclerosis and also of cancer, asthma, hypertension and coughs. F3 and emodin reduced the expression of EBV IE proteins, Rta (R transactivator, the product of *BZLF1* gene), Zta (Z EB replication activator, the product of *BZLF1*) and EA-D (Early Diffuse Protein) in a dose-dependent manner, suggesting that they interfere with an early step of the EBV replication cycle [95]. F3 and emodin inhibited also the *BRLF1* and *BZLF1* mRNA expression, which in turn, affected viral lytic proteins expression and EBV DNA replication. As previous studies have shown that emodin inhibits the activation of p38, MAPK, ERK and JNK signaling and affects the activation of the promoters that are activated by AP-1 and ATF1 [96,97,98], it was suggested that the inhibition of emodin on activation of signaling pathways may be involved in the inhibition of F3 and emodin on the EBV lytic cycle [95]. Inhibition of spontaneous EBV lytic infection by (−)-Epigallocatechin-3-gallate (EGCG), the major green tea catechin, appears to involve also the suppression of activation of MEK/ERK1/2 and PI3-K/Akt signaling [99]. (−)-Green tea is characterized by the presence of high amounts of polyphenolic compounds known as flavanols or catechins and EGCG appears to be the primary active ingredient responsible for the biological effects of green tea, including inhibition of EBV lytic cycle [100].

Recently, the anti-EBV lytic replication activity of lignans isolated from the roots of *Saururus chinensis* was reported [101]. Lignans are the main active constituents of *S. chinensis* and are known to display a broad spectrum of biological activities including NF-kB [102] and HIV protease [103] inhibitor activities and cardiovascular effects [104]. Among 19 new and nine known lignans isolated following fractionation of ethanol extracts of *S. chinensis*, manassantin B exhibited the most promising inhibition with an EC_50_ of 1.72 µM and low toxicity (CC_50_ > 200 µM) and a SI > 116.

Moronic acid, a triterpenoid keto acid, is found in galls of *Rhus chinensis* and Brazilian propolis. Moronic acid was demonstrated to have activity against HIV [105] and EBV [106] in vitro and against HSV-1 in mice [107]. Chang and coworkers reported that moronic acid inhibits the expression of Rta, Zta and EA-D [106]. Furthermore, moronic acid inhibited the capacity of Rta to activate promoters that contain an Rta-response element, indicating that moronic acid interferes with the function of Rta. On the other hand, moronic acid was found to influence the transactivation function of Zta. Hence, the lack of expression of Zta and EA-D following moronic acid treatment could be attributed to the inhibition of the transactivation functions of Rta which results in a substantial reduction in the number of EBV particles produced during the lytic cycle. In contrast, protoapigenone, a flavonoid present in *Thelypteris torresiana*, was reported to inhibit the transactivation function of Zta preventing the virus lytic cycle and to have no impact on the functions of the Rta promoter [108].

Following screening of a laboratory collection of 116 compounds isolated from diverse natural products, angelicin showed antiviral activity against MHV-68 and the two human γ-herpesviruses EBV and KSHV [109]. Angelicin, a furocoumarin present in the seeds of *Psoralea corylifolia* and the roots of *Angelica archangelica*, belongs to the class of psoralens (photo-synthesizers used for the treatment of various skin diseases together with long wavelength UV irradiation). Although detailed molecular mechanisms regarding the mode of action of angelicin against γ-herpesviruses is missing, angelicin was found to inhibit autoactivation of the RTA promoter resulting in inhibition of the early steps of the replicative lytic cycle [109]. Furocoumarins have also proven active against influenza virus [110] and retroviruses [111].

## 8. Use of Antivirals in Lytic Induction Therapy

The development of strategies that reactivate viral lytic replication in latently infected tumor cells (lytic induction therapy) are of increasing interest as lytic replication promotes the death of tumor cells. In addition, tumor cells carrying the virus may also be killed by antiviral drugs (e.g., ganciclovir) which are activated by viral kinases expressed during the lytic cycle. The combination of antiviral agents with inducers of the lytic cycle is being considered as a promising strategy to treat EBV- and KSHV-driven tumors [112,113,114,115]. Further research is required to improve the efficiency of induction to lytic cycle as some cell lines are particularly resistant to lytic activation by external stimuli and even in cell lines that are responsive to lytic induction stimuli, a subpopulation of cells remain unresponsive to lytic cycle activation [112,116].

## 9. Conclusions and Perspectives

Anti-EBV therapy remains a major unmet medical need, in particular for patients with an impaired immune system. Antivirals approved for other herpesviruses that have been evaluated for EBV-associated diseases have delivered disappointing results. A few candidate anti-EBV drugs are available but much work remains to be done to show their efficacy. Further research is needed to develop therapeutic strategies for EBV-associated diseases as well as molecules that could be used in prophylaxis among immunosuppressed patients to avoid complications related to EBV disease. Although an EBV vaccine should be of high benefit to reduce the substantial burden due to primary EBV infection and to diminish the incidence of certain human malignancies, the development of an EBV vaccine has been extremely slow.

A novel strategy that could potentially be used to combat both productive and latent EBV infections is the targeting of viral genetic elements required for viral fitness by CRISPR/Cas9 genome editing techniques. Lebbink’s group demonstrated that by simultaneous targeting of EBV genome with multiple guided RNAs (gRNAs), almost complete clearance of the virus from latently infected EBV-transformed cells was achieved. This opens new avenues for the development of therapeutic approaches to manage pathogenic human herpesviruses by means of novel genome-engineering technologies [117].

## Figures and Tables

**Table 1:** Epstein–Barr (EBV)-associated lymphomas in immunocompetent and immunocompromised hosts.

Immunocompetent Host			Immunocompromised Hosts		
Lymphoma	EBV Association	Latency Program	Lymphoma	EBV Association	Latency Program
BL (endemic)	100%	I or Wp- restricted	PTLD, B-cell	>90%	III
BL (sporadic)	15–85%	I	BL (HIV)	25–35%	I
Classical HL	40%	II	HL (HIV)	>80%	II
DLBCL associated with chronic inflammation	~70%	II	PEL (primary effusion lymphoma)	>80%	I
EBV-positive DLBCL of the elderly	100%	II	Plasmablastic lymphoma	~70%	I or II
Lymphomatoid granulomatosis	100%	II	Plasmablastic lymphoma, oral type (HIV)	100%	I
Angioimmunoblastic T-cell lymphoma *	>90%	II	Primary CNS lymphoma (HIV)	100%	III
Extranodal NK/T-cell lymphoma, nasal type *	100%	II	NHLs with primary immune disorders	>90%	III
Aggressive NK-cell leukemia *	>90%	II	Iatrogenic immunodeficiency lymphoma	40–50%	III
			PTLD, NK/T-cell *	>70%	III

Adapted from [2,3,4,5]. * EBV-associated T- and NK-cell lymphomas. DLBCL: diffuse large B-cell lymphoma. For sporadic Burkitt’s lymphoma (BL), the strength of the EBV association varies with geographical location, hence the wide percentage range reported.

## References

[1] Katz B.Z., Shiraishi Y., Mears C.J., Binns H.J., Taylor R. (2009). Chronic fatigue syndrome after infectious mononucleosis in adolescents. Pediatrics.

[2] Cesarman E. (2011). Gammaherpesvirus and lymphoproliferative disorders in immunocompromised patients. Cancer Lett..

[3] Cesarman E. (2014). Gammaherpesviruses and lymphoproliferative disorders. Annu. Rev. Pathol..

[4] Murata T., Tsurumi T. (2014). Switching of EBV cycles between latent and lytic states. Rev. Med. Virol..

[5] Ryan J.L., Morgan D.R., Dominguez R.L., Thorne L.B., Elmore S.H., Mino-Kenudson M., Lauwers G.Y., Booker J.K., Gulley M.L. (2009). High levels of Epstein–Barr virus DNA in latently infected gastric adenocarcinoma. Lab. Invest..

[6] Miller G., El Guindy A., Countryman J., Ye J., Gradoville L. (2007). Lytic cycle switches of oncogenic human gammaherpesviruses. Adv. Cancer Res..

[7] Heslop H.E. (2009). How I treat EBV lymphoproliferation. Blood.

[8] Hong G.K., Gulley M.L., Feng W.H., Delecluse H.J., Holley-Guthrie E., Kenney S.C. (2005). Epstein–Barr virus lytic infection contributes to lymphoproliferative disease in a SCID mouse model. J. Virol..

[9] Ma S.D., Hegde S., Young K.H., Sullivan R., Rajesh D., Zhou Y., Jankowska-Gan E., Burlingham W.J., Sun X., Gulley M.L. (2011). A new model of Epstein–Barr virus infection reveals an important role for early lytic viral protein expression in the development of lymphomas. J. Virol..

[10] Poole C.L., James S.H. (2018). Antiviral Therapies for Herpesviruses: Current Agents and New Directions. Clin. Ther..

[11] Keith K.A., Hartline C.B., Bowlin T.L., Prichard M.N. (2018). A standardized approach to the evaluation of antivirals against DNA viruses: Polyomaviruses and lymphotropic herpesviruses. Antiviral Res..

[12] Coen N., Duraffour S., Naesens L., Krecmerova M., Van den O.J., Snoeck R., Andrei G. (2013). Evaluation of novel acyclic nucleoside phosphonates against human and animal gammaherpesviruses revealed an altered metabolism of cyclic prodrugs upon Epstein–Barr virus reactivation in P3HR-1 cells. J. Virol..

[13] Coen N., Duraffour S., Topalis D., Snoeck R., Andrei G. (2014). Spectrum of activity and mechanisms of resistance of various nucleoside derivatives against gammaherpesviruses. Antimicrob. Agents Chemother..

[14] Gershburg E., Pagano J.S. (2005). Epstein–Barr virus infections: Prospects for treatment. J. Antimicrob. Chemother..

[15] Ernberg I., Andersson J. (1986). Acyclovir efficiently inhibits oropharyngeal excretion of Epstein–Barr virus in patients with acute infectious mononucleosis. J. Gen. Virol..

[16] Tynell E., Aurelius E., Brandell A., Julander I., Wood M., Yao Q.Y., Rickinson A., Akerlund B., Andersson J. (1996). Acyclovir and prednisolone treatment of acute infectious mononucleosis: A multicenter, double-blind, placebo-controlled study. J. Infect. Dis..

[17] Negro F. (2006). The paradox of Epstein–Barr virus-associated hepatitis. J. Hepatol..

[18] Drebber U., Kasper H.U., Krupacz J., Haferkamp K., Kern M.A., Steffen H.M., Quasdorff M., Zur Hausen A., Odenthal M., Dienes H.P. (2006). The role of Epstein–Barr virus in acute and chronic hepatitis. J. Hepatol..

[19] Kimura H., Cohen J.I. (2017). Chronic Active Epstein–Barr Virus Disease. Front. Immunol..

[20] Okuno Y., Murata T., Sato Y., Muramatsu H., Ito Y., Watanabe T., Okuno T., Murakami N., Yoshida K., Sawada A. (2019). Defective Epstein–Barr virus in chronic active infection and haematological malignancy. Nat. Microbiol..

[21] Wass M., Bauer M., Pfannes R., Lorenz K., Odparlik A., Muller L.P., Wickenhauser C. (2018). Chronic active Epstein–Barr virus infection of T-cell type, systemic form in an African migrant: Case report and review of the literature on diagnostics standards and therapeutic options. BMC Cancer.

[22] Petrara M.R., Giunco S., Serraino D., Dolcetti R., De R.A. (2015). Post-transplant lymphoproliferative disorders: From epidemiology to pathogenesis-driven treatment. Cancer Lett..

[23] Dharnidharka V.R., Webster A.C., Martinez O.M., Preiksaitis J.K., Leblond V., Choquet S. (2016). Post-transplant lymphoproliferative disorders. Nat. Rev. Dis. Primers.

[24] Dierickx D., Habermann T.M. (2018). Post-Transplantation Lymphoproliferative Disorders in Adults. N. Engl. J. Med..

[25] Das B., Morrow R., Huang R., Fixler D. (2016). Persistent Epstein–Barr viral load in Epstein–Barr viral naive pediatric heart transplant recipients: Risk of late-onset post-transplant lymphoproliferative disease. World J. Transplant..

[26] Colombini E., Guzzo I., Morolli F., Longo G., Russo C., Lombardi A., Merli P., Barzon L., Murer L., Piga S. (2017). Viral load of EBV DNAemia is a predictor of EBV-related post-transplant lymphoproliferative disorders in pediatric renal transplant recipients. Pediatr. Nephrol..

[27] Hocker B., Bohm S., Fickenscher H., Kusters U., Schnitzler P., Pohl M., John U., Kemper M.J., Fehrenbach H., Wigger M. (2012). (Val-)Ganciclovir prophylaxis reduces Epstein–Barr virus primary infection in pediatric renal transplantation. Transpl. Int..

[28] Malouf M.A., Chhajed P.N., Hopkins P., Plit M., Turner J., Glanville A.R. (2002). Anti-viral prophylaxis reduces the incidence of lymphoproliferative disease in lung transplant recipients. J. Heart Lung Transplant..

[29] Hierro L., Diez-Dorado R., Diaz C., De L., Frauca E., Camarena C., Munoz-Bartolo G., Gonzalez D.Z., Lopez S.M., Jara P. (2008). Efficacy and safety of valganciclovir in liver-transplanted children infected with Epstein–Barr virus. Liver Transpl..

[30] Cohen M., Vistarop A.G., Huaman F., Narbaitz M., Metrebian F., De Matteo E., Preciado M.V., Chabay P.A. (2018). Epstein–Barr virus lytic cycle involvement in diffuse large B cell lymphoma. Hematol. Oncol..

[31] Jones R.J., Seaman W.T., Feng W.H., Barlow E., Dickerson S., Delecluse H.J., Kenney S.C. (2007). Roles of lytic viral infection and IL-6 in early versus late passage lymphoblastoid cell lines and EBV-associated lymphoproliferative disease. Int. J. Cancer.

[32] Hong G.K., Kumar P., Wang L., Damania B., Gulley M.L., Delecluse H.J., Polverini P.J., Kenney S.C. (2005). Epstein–Barr virus lytic infection is required for efficient production of the angiogenesis factor vascular endothelial growth factor in lymphoblastoid cell lines. J. Virol..

[33] Beatty P.R., Krams S.M., Martinez O.M. (1997). Involvement of IL-10 in the autonomous growth of EBV-transformed B cell lines. J. Immunol..

[34] Coen N., Duraffour S., Haraguchi K., Balzarini J., van den Oord J.J., Snoeck R., Andrei G. (2014). Antiherpesvirus activities of two novel 4’-thiothymidine derivatives, KAY-2-41 and KAH-39-149, are dependent on viral and cellular thymidine kinases. Antimicrob. Agents Chemother..

[35] Coen N., Singh U., Vuyyuru V., Van den Oord J.J., Balzarini J., Duraffour S., Snoeck R., Cheng Y.C., Chu C.K., Andrei G. (2013). Activity and mechanism of action of HDVD, a novel pyrimidine nucleoside derivative with high levels of selectivity and potency against gammaherpesviruses. J. Virol..

[36] Ville S., Imbert-Marcille B.M., Coste-Burel M., Garandeau C., Meurette A., Cantarovitch D., Giral M., Hourmant M., Blancho G., Dantal J. (2018). Impact of antiviral prophylaxis in adults Epstein–Barr Virus-seronegative kidney recipients on early and late post-transplantation lymphoproliferative disorder onset: A retrospective cohort study. Transpl. Int..

[37] Østensen A.B., Sanengen T., Holter E., Line P.D., Almaas R. (2017). No effect of treatment with intravenous ganciclovir on Epstein–Barr virus viremia demonstrated after pediatric liver transplantation. Pediatr. Transplant..

[38] Gill H., Hwang Y.Y., Chan T.S., Pang A.W., Leung A.Y., Tse E., Kwong Y.L. (2014). Valganciclovir suppressed Epstein Barr virus reactivation during immunosuppression with alemtuzumab. J. Clin. Virol..

[39] Yager J.E., Magaret A.S., Kuntz S.R., Selke S., Huang M.L., Corey L., Casper C., Wald A. (2017). Valganciclovir for the Suppression of Epstein–Barr Virus Replication. J. Infect. Dis..

[40] Snoeck R., De Clercq E. (2002). Role of cidofovir in the treatment of DNA virus infections, other than CMV infections, in immunocompromised patients. Curr. Opin. Investig. Drugs.

[41] Andrei G., Topalis D., De Schutter T., Snoeck R. (2015). Insights into the mechanism of action of cidofovir and other acyclic nucleoside phosphonates against polyoma- and papillomaviruses and non-viral induced neoplasia. Antiviral. Res..

[42] Yoshizaki T., Wakisaka N., Kondo S., Murono S., Shimizu Y., Nakashima M., Tsuji A., Furukawa M. (2008). Treatment of locally recurrent Epstein–Barr virus-associated nasopharyngeal carcinoma using the anti-viral agent cidofovir. J. Med. Virol..

[43] Neyts J., Sadler R., De Clercq E., Raab-Traub N., Pagano J.S. (1998). The antiviral agent cidofovir [(*S*)-1-(3-hydroxy-2-phosphonyl-methoxypropyl)cytosine] has pronounced activity against nasopharyngeal carcinoma grown in nude mice. Cancer Res..

[44] Wakisaka N., Yoshizaki T., Raab-Traub N., Pagano J.S. (2005). Ribonucleotide reductase inhibitors enhance cidofovir-induced apoptosis in EBV-positive nasopharyngeal carcinoma xenografts. Int. J. Cancer.

[45] Abdulkarim B., Sabri S., Zelenika D., Deutsch E., Frascogna V., Klijanienko J., Vainchenker W., Joab I., Bourhis J. (2003). Antiviral agent cidofovir decreases Epstein–Barr virus (EBV) oncoproteins and enhances the radiosensitivity in EBV-related malignancies. Oncogene.

[46] Afshar K., Rao A.P., Patel V., Forrester K., Ganesh S. (2011). Use of Foscarnet Therapy for EBV Infection following Control of PTLD with Enhancement of Cellular Immunity in a Lung-Transplant Recipient. J. Transplant..

[47] Schneider U., Ruhnke M., Delecluse H.J., Stein H., Huhn D. (2000). Regression of Epstein–Barr virus-associated lymphoproliferative disorders in patients with acquired immunodeficiency syndrome during therapy with foscarnet. Ann. Hematol..

[48] Biron K.K., Harvey R.J., Chamberlain S.C., Good S.S., Smith A.A., Davis M.G., Talarico C.L., Miller W.H., Ferris R., Dornsife R.E. (2002). Potent and selective inhibition of human cytomegalovirus replication by 1263W94, a benzimidazole L-riboside with a unique mode of action. Antimicrob. Agents Chemother..

[49] Zacny V.L., Gershburg E., Davis M.G., Biron K.K., Pagano J.S. (1999). Inhibition of Epstein–Barr virus replication by a benzimidazole L-riboside: Novel antiviral mechanism of 5, 6-dichloro-2-(isopropylamino)-1-beta-L-ribofuranosyl-1H-benzimidazole. J. Virol..

[50] Prichard M.N. (2009). Function of human cytomegalovirus UL97 kinase in viral infection and its inhibition by maribavir. Rev. Med. Virol..

[51] Chou S., Bowlin T.L. (2011). Cytomegalovirus UL97 mutations affecting cyclopropavir and ganciclovir susceptibility. Antimicrob. Agents Chemother..

[52] Sharma M., Bender B.J., Kamil J.P., Lye M.F., Pesola J.M., Reim N.I., Hogle J.M., Coen D.M. (2014). Human Cytomegalovirus UL97 Phosphorylates the Viral Nuclear Egress Complex. J. Virol..

[53] Lurain N.S., Chou S. (2010). Antiviral drug resistance of human cytomegalovirus. Clin. Microbiol. Rev..

[54] Chou S. (2008). Cytomegalovirus UL97 mutations in the era of ganciclovir and maribavir. Rev. Med. Virol..

[55] Chou S., Marousek G., Bowlin T.L. (2012). Cyclopropavir susceptibility of cytomegalovirus DNA polymerase mutants selected after antiviral drug exposure. Antimicrob. Agents Chemother..

[56] Chou S., Ercolani R.J., Marousek G., Bowlin T.L. (2013). Cytomegalovirus UL97 kinase catalytic domain mutations that confer multidrug resistance. Antimicrob. Agents Chemother..

[57] Winston D.J., Saliba F., Blumberg E., Abouljoud M., Garcia-Diaz J.B., Goss J.A., Clough L., Avery R., Limaye A.P., Ericzon B.G. (2012). Efficacy and safety of maribavir dosed at 100 mg orally twice daily for the prevention of cytomegalovirus disease in liver transplant recipients: A randomized, double-blind, multicenter controlled trial. Am. J. Transplant..

[58] Marty F.M., Ljungman P., Papanicolaou G.A., Winston D.J., Chemaly R.F., Strasfeld L., Young J.A., Rodriguez T., Maertens J., Schmitt M. (2011). Maribavir prophylaxis for prevention of cytomegalovirus disease in recipients of allogeneic stem-cell transplants: A phase 3, double-blind, placebo-controlled, randomised trial. Lancet. Infect. Dis..

[59] Griffiths P., Lumley S. (2014). Cytomegalovirus. Curr. Opin. Infect. Dis..

[60] Wang F.Z., Roy D., Gershburg E., Whitehurst C.B., Dittmer D.P., Pagano J.S. (2009). Maribavir inhibits Epstein–Barr virus transcription in addition to viral DNA replication. J. Virol..

[61] Murata T., Isomura H., Yamashita Y., Toyama S., Sato Y., Nakayama S., Kudoh A., Iwahori S., Kanda T., Tsurumi T. (2009). Efficient production of infectious viruses requires enzymatic activity of Epstein–Barr virus protein kinase. Virology.

[62] Whitehurst C.B., Sanders M.K., Law M., Wang F.Z., Xiong J., Dittmer D.P., Pagano J.S. (2013). Maribavir inhibits Epstein–Barr virus transcription through the EBV protein kinase. J. Virol..

[63] Meng Q., Hagemeier S.R., Fingeroth J.D., Gershburg E., Pagano J.S., Kenney S.C. (2010). The Epstein–Barr virus (EBV)-encoded protein kinase, EBV-PK, but not the thymidine kinase (EBV-TK), is required for ganciclovir and acyclovir inhibition of lytic viral production. J. Virol..

[64] Qiu Y.L., Geiser F., Kira T., Gullen E., Cheng Y.C., Ptak R.G., Breitenbach J.M., Drach J.C., Hartline C.B., Kern E.R. (2000). Synthesis and enantioselectivity of the antiviral effects of (R,Z)-,(S,Z)-methylenecyclopropane analogues of purine nucleosides and phosphoralaninate prodrugs: Influence of heterocyclic base, type of virus and host cells. Antivir. Chem. Chemother..

[65] Zhou S., Breitenbach J.M., Borysko K.Z., Drach J.C., Kern E.R., Gullen E., Cheng Y.C., Zemlicka J. (2004). Synthesis and antiviral activity of (*Z*)- and (*E*)-2,2-[bis(hydroxymethyl)cyclopropylidene]methylpurines and -pyrimidines: Second-generation methylenecyclopropane analogues of nucleosides. J. Med. Chem..

[66] Prichard M.N., Williams J.D., Komazin-Meredith G., Khan A.R., Price N.B., Jefferson G.M., Harden E.A., Hartline C.B., Peet N.P., Bowlin T.L. (2013). Synthesis and antiviral activities of methylenecyclopropane analogs with 6-alkoxy and 6-alkylthio substitutions that exhibit broad-spectrum antiviral activity against human herpesviruses. Antimicrob. Agents Chemother..

[67] Chen X., Kern E.R., Drach J.C., Gullen E., Cheng Y.C., Zemlicka J. (2003). Structure-activity relationships of (*S*,*Z*)-2-aminopurine methylenecyclopropane analogues of nucleosides. Variation of purine-6 substituents and activity against herpesviruses and hepatitis B virus. J. Med. Chem..

[68] Kern E.R., Kushner N.L., Hartline C.B., Williams-Aziz S.L., Harden E.A., Zhou S., Zemlicka J., Prichard M.N. (2005). In vitro activity and mechanism of action of methylenecyclopropane analogs of nucleosides against herpesvirus replication. Antimicrob. Agents Chemother..

[69] Li C., Quenelle D.C., Prichard M.N., Drach J.C., Zemlicka J. (2012). Synthesis and antiviral activity of 6-deoxycyclopropavir, a new prodrug of cyclopropavir. Bioorg. Med. Chem..

[70] James S.H., Hartline C.B., Harden E.A., Driebe E.M., Schupp J.M., Engelthaler D.M., Keim P.S., Bowlin T.L., Kern E.R., Prichard M.N. (2011). Cyclopropavir inhibits the normal function of the human cytomegalovirus UL97 kinase. Antimicrob. Agents Chemother..

[71] Prichard M.N., Keith K.A., Quenelle D.C., Kern E.R. (2006). Activity and mechanism of action of *N*-methanocarbathymidine against herpesvirus and orthopoxvirus infections. Antimicrob. Agents Chemother..

[72] Quenelle D.C., Collins D.J., Rice T.L., Rahman A., Glazer R. (2011). Efficacy of orally administered low dose *N*-methanocarbathymidine against lethal herpes simplex virus type-2 infections of mice. Antivir. Chem. Chemother..

[73] Bernstein D.I., Bravo F.J., Pullum D.A., Shen H., Wang M., Rahman A., Glazer R.I., Cardin R.D. (2015). Efficacy of N-methanocarbathymidine against genital herpes simplex virus type 2 shedding and infection in guinea pigs. Antivir. Chem. Chemother..

[74] Hostetler K.Y. (2009). Alkoxyalkyl prodrugs of acyclic nucleoside phosphonates enhance oral antiviral activity and reduce toxicity: Current state of the art. Antiviral. Res..

[75] Marty F.M., Winston D.J., Chemaly R.F., Mullane K.M., Shore T.B., Papanicolaou G.A., Chittick G., Brundage T.M., Wilson C., Morrison M.E. (2019). A Randomized, Double-Blind, Placebo-Controlled Phase 3 Trial of Oral Brincidofovir for Cytomegalovirus Prophylaxis in Allogeneic Hematopoietic Cell Transplantation. Biol. Blood Marrow Transplant..

[76] Wilson J.B., Manet E., Gruffat H., Busson P., Blondel M., Fahraeus R. (2018). EBNA1: Oncogenic Activity, Immune Evasion and Biochemical Functions Provide Targets for Novel Therapeutic Strategies against Epstein–Barr Virus- Associated Cancers. Cancers.

[77] Jiang L., Xie C., Lung H.L., Lo K.W., Law G.L., Mak N.K., Wong K.L. (2018). EBNA1-targeted inhibitors: Novel approaches for the treatment of Epstein–Barr virus-associated cancers. Theranostics.

[78] Lee E.K., Kim S.Y., Noh K.W., Joo E.H., Zhao B., Kieff E., Kang M.S. (2014). Small molecule inhibition of Epstein–Barr virus nuclear antigen-1 DNA binding activity interferes with replication and persistence of the viral genome. Antiviral. Res..

[79] Gianti E., Messick T.E., Lieberman P.M., Zauhar R.J. (2016). Computational analysis of EBNA1 “druggability” suggests novel insights for Epstein–Barr virus inhibitor design. J. Comput. Aided Mol. Des..

[80] Kawanishi M. (1993). Topoisomerase I and II activities are required for Epstein–Barr virus replication. J. Gen. Virol..

[81] Wang P., Rennekamp A.J., Yuan Y., Lieberman P.M. (2009). Topoisomerase I and RecQL1 function in Epstein–Barr virus lytic reactivation. J. Virol..

[82] Wu T., Wang Y., Yuan Y. (2014). Antiviral activity of topoisomerase II catalytic inhibitors against Epstein–Barr virus. Antiviral. Res..

[83] Gonzalez-Molleda L., Wang Y., Yuan Y. (2012). Potent antiviral activity of topoisomerase I and II inhibitors against Kaposi’s sarcoma-associated herpesvirus. Antimicrob. Agents Chemother..

[84] Widman D.G., Gornisiewicz S., Shacham S., Tamir S. (2018). In vitro toxicity and efficacy of verdinexor, an exportin 1 inhibitor, on opportunistic viruses affecting immunocompromised individuals. PLoS ONE.

[85] Li R., Hayward S.D. (2013). Potential of protein kinase inhibitors for treating herpesvirus-associated disease. Trends Microbiol..

[86] Vigano M., Dengler T., Mattei M.F., Poncelet A., Vanhaecke J., Vermes E., Kleinloog R., Li Y., Gezahegen Y., Delgado J.F. (2010). Lower incidence of cytomegalovirus infection with everolimus versus mycophenolate mofetil in de novo cardiac transplant recipients: A randomized, multicenter study. Transpl. Infect. Dis..

[87] Hill J.A., Hummel M., Starling R.C., Kobashigawa J.A., Perrone S.V., Arizon J.M., Simonsen S., Abeywickrama K.H., Bara C. (2007). A lower incidence of cytomegalovirus infection in de novo heart transplant recipients randomized to everolimus. Transplantation.

[88] Kobashigawa J., Ross H., Bara C., Delgado J.F., Dengler T., Lehmkuhl H.B., Wang S.S., Dong G., Witte S., Junge G. (2013). Everolimus is associated with a reduced incidence of cytomegalovirus infection following de novo cardiac transplantation. Transpl. Infect. Dis..

[89] Tan L., Sato N., Shiraki A., Yanagita M., Yoshida Y., Takemura Y., Shiraki K. (2019). Everolimus delayed and suppressed cytomegalovirus DNA synthesis, spread of the infection, and alleviated cytomegalovirus infection. Antiviral. Res..

[90] Lin T.P., Chen S.Y., Duh P.D., Chang L.K., Liu Y.N. (2008). Inhibition of the Epstein–Barr virus lytic cycle by andrographolide. Biol. Pharm. Bull..

[91] Uttekar M.M., Das T., Pawar R.S., Bhandari B., Menon V., Nutan, Gupta S.K., Bhat S.V. (2012). Anti-HIV activity of semisynthetic derivatives of andrographolide and computational study of HIV-1 gp120 protein binding. Eur. J. Med. Chem..

[92] Aromdee C., Suebsasana S., Ekalaksananan T., Pientong C., Thongchai S. (2011). Stage of action of naturally occurring andrographolides and their semisynthetic analogues against herpes simplex virus type 1 in vitro. Planta Med..

[93] Zhou B., Zhang D., Wu X. (2013). Biological activities and corresponding SARs of andrographolide and its derivatives. Mini. Rev. Med. Chem..

[94] Wiart C., Kumar K., Yusof M.Y., Hamimah H., Fauzi Z.M., Sulaiman M. (2005). Antiviral properties of ent-labdene diterpenes of Andrographis paniculata nees, inhibitors of herpes simplex virus type 1. Phytother. Res..

[95] Yiu C.Y., Chen S.Y., Yang T.H., Chang C.J., Yeh D.B., Chen Y.J., Lin T.P. (2014). Inhibition of Epstein–Barr virus lytic cycle by an ethyl acetate subfraction separated from Polygonum cuspidatum root and its major component, emodin. Molecules.

[96] Li D., Zhang N., Cao Y., Zhang W., Su G., Sun Y., Liu Z., Li F., Liang D., Liu B. (2013). Emodin ameliorates lipopolysaccharide-induced mastitis in mice by inhibiting activation of NF-kappaB and MAPKs signal pathways. Eur. J. Pharmacol..

[97] Lee J., Jung E., Lee J., Huh S., Hwang C.H., Lee H.Y., Kim E.J., Cheon J.M., Hyun C.G., Kim Y.S. (2006). Emodin inhibits TNF alpha-induced MMP-1 expression through suppression of activator protein-1 (AP-1). Life Sci..

[98] Lin H.J., Chao P.D., Huang S.Y., Wan L., Wu C.J., Tsai F.J. (2007). Aloe-emodin suppressed NMDA-induced apoptosis of retinal ganglion cells through regulation of ERK phosphorylation. Phytother. Res..

[99] Liu S., Li H., Chen L., Yang L., Li L., Tao Y., Li W., Li Z., Liu H., Tang M. (2013). (−)-Epigallocatechin-3-gallate inhibition of Epstein–Barr virus spontaneous lytic infection involves ERK1/2 and PI3-K/Akt signaling in EBV-positive cells. Carcinogenesis.

[100] Chang L.K., Wei T.T., Chiu Y.F., Tung C.P., Chuang J.Y., Hung S.K., Li C., Liu S.T. (2003). Inhibition of Epstein–Barr virus lytic cycle by (−)-epigallocatechin gallate. Biochem. Biophys. Res. Commun..

[101] Cui H., Xu B., Wu T., Xu J., Yuan Y., Gu Q. (2014). Potential antiviral lignans from the roots of Saururus chinensis with activity against Epstein–Barr virus lytic replication. J. Nat. Prod..

[102] Hwang B.Y., Lee J.H., Nam J.B., Hong Y.S., Lee J.J. (2003). Lignans from Saururus chinensis inhibiting the transcription factor NF-kappaB. Phytochemistry.

[103] Lee J., Huh M.S., Kim Y.C., Hattori M., Otake T. (2010). Lignan, sesquilignans and dilignans, novel HIV-1 protease and cytopathic effect inhibitors purified from the rhizomes of Saururus chinensis. Antiviral. Res..

[104] Oh K.S., Choi Y.H., Ryu S.Y., Oh B.K., Seo H.W., Yon G.H., Kim Y.S., Lee B.H. (2008). Cardiovascular effects of lignans isolated from Saururus chinensis. Planta Med..

[105] Yu D., Sakurai Y., Chen C.H., Chang F.R., Huang L., Kashiwada Y., Lee K.H. (2006). Anti-AIDS agents 69. Moronic acid and other triterpene derivatives as novel potent anti-HIV agents. J. Med. Chem..

[106] Chang F.R., Hsieh Y.C., Chang Y.F., Lee K.H., Wu Y.C., Chang L.K. (2010). Inhibition of the Epstein–Barr virus lytic cycle by moronic acid. Antiviral. Res..

[107] Kurokawa M., Basnet P., Ohsugi M., Hozumi T., Kadota S., Namba T., Kawana T., Shiraki K. (1999). Anti-herpes simplex virus activity of moronic acid purified from Rhus javanica in vitro and in vivo. J. Pharmacol. Exp. Ther..

[108] Tung C.P., Chang F.R., Wu Y.C., Chuang D.W., Hunyadi A., Liu S.T. (2011). Inhibition of the Epstein–Barr virus lytic cycle by protoapigenone. J. Gen. Virol..

[109] Cho H.J., Jeong S.G., Park J.E., Han J.A., Kang H.R., Lee D., Song M.J. (2013). Antiviral activity of angelicin against gammaherpesviruses. Antiviral. Res..

[110] Yeh J.Y., Coumar M.S., Horng J.T., Shiao H.Y., Kuo F.M., Lee H.L., Chen I.C., Chang C.W., Tang W.F., Tseng S.N. (2010). Anti-influenza drug discovery: Structure-activity relationship and mechanistic insight into novel angelicin derivatives. J. Med. Chem..

[111] Miolo G., Tomanin R., De Rossi A., Dall’Acqua F., Zacchello F., Scarpa M. (1994). Antiretroviral activity of furocoumarins plus UVA light detected by a replication-defective retrovirus. J. Photochem. Photobiol..

[112] Gorres K.L., Daigle D., Mohanram S., Miller G. (2014). Activation and repression of Epstein–Barr Virus and Kaposi’s sarcoma-associated herpesvirus lytic cycles by short- and medium-chain fatty acids. J. Virol..

[113] Hui K., Ho D.N., Tsang C., Middeldorp J.M., Tsao G.S., Chiang A.K. (2012). Activation of lytic cycle of Epstein–Barr virus by suberoylanilide hydroxamic acid leads to apoptosis and tumor growth suppression of nasopharyngeal carcinoma. Int. J. Cancer.

[114] Tang W., Harmon P., Gulley M.L., Mwansambo C., Kazembe P.N., Martinson F., Wokocha C., Kenney S.C., Hoffman I., Sigel C. (2010). Viral response to chemotherapy in endemic burkitt lymphoma. Clin. Cancer Res..

[115] Fu D.X., Tanhehco Y., Chen J., Foss C.A., Fox J.J., Chong J.M., Hobbs R.F., Fukayama M., Sgouros G., Kowalski J. (2008). Bortezomib-induced enzyme-targeted radiation therapy in herpesvirus-associated tumors. Nat. Med..

[116] Countryman J.K., Gradoville L., Miller G. (2008). Histone hyperacetylation occurs on promoters of lytic cycle regulatory genes in Epstein–Barr virus-infected cell lines which are refractory to disruption of latency by histone deacetylase inhibitors. J. Virol..

[117] Van Diemen F.R., Kruse E.M., Hooykaas M.J., Bruggeling C.E., Schurch A.C., van Ham P.M., Imhof S.M., Nijhuis M., Wiertz E.J., Lebbink R.J. (2016). CRISPR/Cas9-Mediated Genome Editing of Herpesviruses Limits Productive and Latent Infections. PLoS Pathog..

